# 2015 Review of Newly Approved Oncologic Therapies

**DOI:** 10.6004/jadpro.2016.7.3.4

**Published:** 2016-04-01

**Authors:** Patrick Medina, Monique Giordana

**Affiliations:** University of Oklahoma College of Pharmacy, Oklahoma City, Oklahoma, and Regions Hospital, St. Paul, Minnesota

A number of cancer drugs approved in 2014 and 2015 should advance the care of patients and improve treatement outcomes, according to descriptions of new agents presented by Patrick Medina, PharmD, of the University of Oklahoma College of Pharmacy, Oklahoma City, Oklahoma, and Monique Giordana, PharmD, BCOP, of Regions Hospital, St. Paul, Minnesota, at the JADPRO Live at APSHO conference.

## PATHWAY INHIBITORS

Two molecular pathway inhibitors were approved: sonidegib (Odomzo) and olaparib (Lynparza). Sonidegib, the second oral hedgehog inhibitor, is indicated for adults with locally advanced basal cell carcinoma that has recurred after surgery or radiation therapy or for those ineligible for surgery or radiation. Supporting data came from the randomized phase II BOLT trial (Migden et al., 2015).

Advanced practitioners should be on the alert for the adverse events reported with this oral agent, including muscle spasms, fatigue, musculoskeletal pain, decreased weight, decreased appetite, abdominal pain, and pruritus.

"The majority of patients you put on this drug will get muscle pain," Dr. Medina indicated.

"If your patient develops any new muscle pain, you want to get a creatinine kinase level, and follow the specific recommendations for holding the drug based on elevated levels," he said. "That is a fairly unique side effect for this class of agents, and there are some differences here between this drug and vismodegib, which does not cause as big of an increase in creatinine kinase."

The PARP inhibitor olaparib is indicated as a single-agent treatment for patients with deleterious or suspected deleterious germline BRCA-mutated advanced ovarian cancer who have been treated with three or more prior lines of chemotherapy. Approval was based on a clinical trial in which responses to olaparib were observed across different tumor types with these mutations (Kaufman et al., 2015). 

The main side effects of olaparib are anemia, nausea, vomiting, dyspepsia, upper respiratory infection, arthralgia, and rash. "Although anemia is the biggest side effect, arthralgia can be troublesome for patients," Dr. Giordana noted.

## IMMUNOTHERAPIES

The treatment of advanced melanoma has been dramatically improved by the anti–programmed cell death protein 1 (anti–PD-1) agents, two of which became available for this malignancy in 2014: nivolumab (Opdivo) and pembrolizumab (Keytruda). In late 2015, these agents were also approved for the treatment of non–small cell lung cancer (NSCLC), based on data for nivolumab from CheckMate-017 and CheckMate-057 (Spigel et al., 2015; Paz-Ares et al., 2015) and for pembrolizumab from the Keynote-001 trial (Garon et al., 2015). 

Elaborating on the success of nivolumab in melanoma, Dr. Giordana noted that the 32% objective response rate seen in the CheckMate-037 trial is a vast improvement over the 11% response rate seen with conventional therapies (Weber et al., 2015). "This is quite remarkable improvement. We have never seen these types of response rates before in melanoma," she said. 

Recent phase III data suggest that by combining pembrolizumab and the CTLA4 inhibitor ipilimumab, response rates can reach 60% in advanced melanoma, she added.

The most common immune-therapy adverse events reported with these agents are fatigue, diarrhea, and skin toxicity with rash.

## OTHER AGENTS

Drs. Medina and Giordana discussed several other new and important agents approved in 2015, some of which are summarized here:

*Gefitinib (Iressa)*: This epidermal growth factor receptor (EGFR) tyrosine kinase inhibitor is indicated only for the first-line treatment of patients with metastatic NSCLC whose tumors have EGFR exon 19 deletions or exon 21 (L858R) substitution mutations. Rash is a frequent toxicity and should be treated aggressively with antibiotics, topical medications and, if grade 2 or higher, systemic steroids.

*Dinutuximab (Unituxin)*: The approval of this chimeric GD2 antibody for children with high-risk neuroblastoma was "huge news in the pediatric world," Dr. Medina said. Based on a 46% reduction in events (Yu et al., 2010), it is indicated in combination with granulocyte-macrophage colony-stimulating factor, interleukin 2, and 13-cis-retinoic acid in high-risk pediatric patients who achieve at least a partial response to prior first-line therapy. Its drawback is the potential for severe neuropathic pain, which makes it difficult to deliver to patients, he added.

*Lenvatinib (Lenvima)*: This vascular endothelial growth factor receptor is indicated for treatment of locally recurrent or metastatic progressive, radioactive iodine–refractory differentiated thyroid cancer. This is a highly effective drug for a "somewhat niche population," as it reduced risk of progression by 79% (Schlumberger et al., 2015), according to Dr. Medina.

*Palbociclib (Ibrance)*: This cyclin-dependent kinase 4 and 6 inhibitor is indicated in combination with letrozole for first-line treatment of postmenopausal women with estrogen receptor–positive, HER2-negative metastatic breast cancer, based on a 51% reduction in risk of progression (Finn et al., 2014). The drawback is a fairly high rate of neutropenia, and interaction with CYP3A inhibitors and inducers. According to Dr. Medina, palbociclib is expected to be useful beyond breast cancer.

Other antitumor agents approved in the past 2 years and now being used in the clinic include blinatumumab for Ph-negative relapsed/refractory B-cell precursor acute lymphoblastic leukemia, panobinostat (plus bortezomib/dexamethasone) in relapsed/refractory multiple myeloma, and the somatostatin analog lanreotide for the re-treatment of gastroenteropancreatic neuroendocrine tumors.
